# Identification of *Bacillus* species: Implication on the quality of probiotic formulations

**DOI:** 10.1371/journal.pone.0217021

**Published:** 2019-05-20

**Authors:** Francesco Celandroni, Alessandra Vecchione, Alice Cara, Diletta Mazzantini, Antonella Lupetti, Emilia Ghelardi

**Affiliations:** 1 Department of Translational Research and New Technologies in Medicine and Surgery, University of Pisa, Pisa, Italy; 2 Research Center Nutraceuticals and Food for Health-Nutrafood, University of Pisa, Pisa, Italy; Chandigarh University, INDIA

## Abstract

Spores of several *Bacillus* species have long history of consumption and safe use as probiotics and a variety of formulations containing these organisms are available in the global market. Considering the difficulties in the identification of *Bacillus* species and the poor microbiological quality of many probiotic formulations, we used three up-to-date methodological approaches for analyzing the content of ten formulations marketed in Italy and labeled to contain *Bacillus* spores. We compared the performance of biochemical tests based on the BCL Vitek2 card and MALDI-TOF mass spectrometry, using 16S rDNA sequencing as the reference technique. The BCL card performed well in identifying all *Bacillus* probiotic strains as well as the Bruker’s MALDI Biotyper. Nevertheless, the MALDI score values were sometimes lower than those indicated by the manufacturer for correct species identification. Contaminant bacteria (*Lysinibacillus fusiformis*, *Acinetobacter baumannii*, *Bacillus cereus*, *Brevibacillus choshinensis*, *Bacillus licheniformis*, *Bacillus badius*) were detected in some formulations. Characterization of the *B*. *cereus* contaminant showed the potential pathogenicity of this strain. Microbial enumeration performed by the plate count method revealed that the number of viable cells contained in many of the analyzed products differed from the labeled amount. Overall, our data show that only two of the ten analyzed formulations qualitatively and quantitatively respect what is on the label. Since probiotic properties are most often strain specific, molecular typing of isolates of the two most common *Bacillus* species, *B*. *clausii* and *B*. *coagulans*, was also performed. In conclusion, the majority of the analyzed products do not comply with quality requirements, most likely leading to reduced/absent efficacy of the preparation and representing a potential infective risk for consumers.

## Introduction

The genus *Bacillus* is a phenotypically large, heterogeneous collection of Gram-positive or Gram-variable spore-forming, aerobic or facultative anaerobic bacteria that have undergone considerable reclassification following the advances in molecular biology techniques [1, 2]. For their wide range of physiologic characteristics and ability to produce a multitude of enzymes, antibiotics, and metabolites, *Bacillus* species are used in many medical, pharmaceutical, agricultural, and industrial processes. Different species produce nutraceuticals such as vitamins (e.g., riboflavin, cobalamin, and inositol) and carotenoids and have been used for the synthesis of several health supplements for human consumption [3–5]. In addition, *Bacillus* spores have a long history of consumption and safe use as probiotics [6], live microorganisms that when administered in adequate amounts confer a health benefit on the host. Probiotic products containing spores for human or animal use are commercialized in several countries, being widespread in Australia, Asia, USA, South America and Europe [6, 7]. Italy has a long story on the use of spore-based probiotics for human consumption, with a *Bacillus clausii* spore suspension being available since 1958 [8].

The identification of species in the genus *Bacillus* by classical methods is often difficult, despite still prevailing in many Microbiology laboratories, due to similarities among closely related species that share a pattern of morphological, biochemical, and genetic characteristics. The use of matrix-assisted laser desorption ionization time-of-flight mass spectrometry (MALDI-TOF MS) as a diagnostic technique for *Bacillus* spp. identification has been applied for addressing the challenges associated with the identification of these organisms [9–15]. However, few data are available on the application of MALDI-TOF MS for the identification of probiotic strains belonging to this genus [16–17].

A number of studies have highlighted the poor microbiological quality of many commercial probiotic formulations in terms of identification, viability, or number of microorganisms [16, 18–26], thus potentially precluding the expected health benefit of the preparation and representing a potential infective risk for consumers. On the other hand, limited data are available on the compositional quality of formulations containing *Bacillus* spores [27, 28].

In this study, we evaluated qualitative and quantitative aspects of ten probiotic formulations marketed in Italy and containing *Bacillus* spores. We compared the performance of MALDI-TOF MS (Bruker Daltonics, Bremen, Germany) and biochemical tests based on the Vitek2 BCL card (bioMeriéux, Marcy l’Etoile, France), using 16S rDNA sequencing as reference technique for the identification of the probiotic spore-formers. Since the beneficial health effects of probiotics may be strain-specific [29], molecular typing of strains belonging to the most common species isolated from different formulations was also applied.

## Materials and methods

### Commercial probiotic formulations and bacterial strains

The formulations analyzed in this study are reported in Table 1. All formulations were purchased in pharmacies by the investigators and investigated before the expiration date. Capsules and lyophilized preparations were dissolved in sterile water immediately before the analyses were performed. Liquid formulations were directly used for analyses. The reference strains *Bacillus cereus* ATCC 14579, *Bacillus clausii* ATCC 10317, *B*. *clausii* ATCC 21536, *B*. *clausii* ATCC 21537, *B*. *clausii* DSM 8716, and two *Bacillus coagulans* from our collection (FLtas1 and FP22) were used as control strains.

**Table 1:** Probiotic products used in this study.

Formulation n.	Product	Brand	Batch
1	Biogermin vials	Union Health S.r.l.	40239
2	Biolactine family bottles	Sella S.r.l.	L096264
3	Enterofermenti family vials	SB Pharma C.	L70316
4	Enterogermina 2 mld vials	Sanofi S.p.A.	1739
5	Enterolife vials	Paladin Pharma S.p.A.	L100516
6	Ferzym Plus capsules	Specchiasol S.r.l.	02427
7	Lactò Più bottles	Recordati OTC S.p.A.	0134
8	Nucleogermina 10 bottles	Pharmaelle S.r.l.	020415
9	Progermila bottles	Chemist’s Research S.r.l.	171214
10	Progermila bambini bottles	Chemist’s Research S.r.l.	140316

Brand and batch of the analyzed formulations.

### Identification of spore-forming probiotic strains

In order to inactivate bacterial vegetative forms, the microbial suspensions of probiotic organisms were thermally treated by exposing to 80°C for 15 min, serially diluted in PBS and seeded (100 μl per plate) on trypticase soy agar with 5% horse blood (TSH, bioMérieux, Marcy l'Etoile, France). Plates were incubated at 37°C in aerobic atmosphere for 24–48 hours and morphologically different colonies were subjected to identification by biochemical analysis using the BCL card of the Vitek2 system (bioMérieux, Marcy l'Etoile, France). This system expresses the identification profiles as the probability (%) of identity between the tested strain and the database taxa. A probability ranging between 96–99% is associated with an excellent confidence level of identification, between 93–95% with a very good level, between 89–92% with a good level, between 85–88% with an acceptable level, and lower probabilities are considered not discriminative. In parallel, bacteria from single colonies were used for Matrix Assisted Laser Desorption Ionization-Time of Flight Mass Spectrometry (MALDI-TOF MS) analysis in a Microflex LT MALDI Biotyper mass spectrometer (Bruker Daltonics, Bremen, Germany). Bacteria were also subjected to identification by 16S rRNA gene sequencing. Experiments were repeated three times in separate days using different doses of the same batches.

In order to separate the four polyantibiotic-resistant *Bacillus* strains (OC, NR, SIN, T) contained in formulation 4, aliquots of the product were seeded onto Mueller Hinton agar plates with different antibiotics: chloramphenicol (50 μgml^-1^), rifampicin (50 μgml^-1^) plus novobiocin (100 μgml^-1^), streptomycin (200 μgml^-1^) plus neomycin (100 μgml^-1^), and tetracycline (100 μgml^-1^) [8].

### MALDI-TOF MS analysis

A colony was directly spotted on the MALDI plate and treated with 1 μl of ethanol, 1 μl of formic acid and 1 μl of acetonitrile for protein extraction, and then overlaid with 1 μl of saturated α-cyano-4-hydroxycinnamic acid (HCCA) matrix solution and air-dried. Each isolate was tested in duplicate. The loaded plate was then placed in the instrument according to the manufacturer’s instructions. The mass spectra were acquired automatically, within 5–10 min, in the positive linear mode at a laser frequency of 60Hz with an acquisition range from 1.960 to 20.000 Da. The spectra were imported into the integrated MALDI Biotyper software (version 3.1) and analyzed by standard pattern matching with a default database.

### 16S rRNA gene sequencing

Genomic DNA was extracted and purified as previously described [30]. A sequence ranging from nucleotide 9 to nucleotide 1523 of the 1556 bp 16S rRNA gene (97.4%) was amplified with the universal primers 27F (5’-GAGAGTTTGATCCTGGCTCAG-3’) and 1495R (5’-CTACGGCTACCTTGTTACGA-3’). Amplified fragments were purified and sequenced using the same primers (Eurofins MWG Operon, Germany). Sequences were compared with those contained in the Ribosomal Database Project. Identity scores of 97% and 99% were considered for the identification at genus and species level respectively, according to the Clinical and Laboratory Standards Institute (CLSI) guidelines [31].

### Bacterial enumeration

Enumeration of spores and viable bacteria was conducted by the plate count method. Microbial suspensions of probiotic organisms were divided into two aliquots and one aliquot was heat-treated at 80°C for 15 min prior to plating. Thermally treated and untreated suspensions were serially diluted in PBS and seeded on TSH. Plating was performed in triplicate and the experiments were repeated three times in separate days using different doses of the same batches. Plates were incubated at 37°C in aerobic atmosphere for 48 h and the number of CFU was determined. Microbial counts were expressed as the mean ± standard deviation.

### Evaluation of *B*. *cereus* virulence potential

Analysis of the presence of genes encoding typical *B*. *cereus* toxins (sphingomyelinase, *sph*; enterotoxin T, *bcet*; enterotoxin FM, *entFM*; enterotoxin S, *entS*; phosphatidylinositol-specific phospholipase C, *plcA*; cytotoxin K, *cytK*; non-hemolytic enterotoxin complex, *nheA*, *nheB*, and *nheC*; component L2 of hemolysin BL *hblC*) was performed by specific PCR reactions as previously described [32, 33]. Phosphatidylcholine-specific phospholipase C (PC-PLC) activity in culture supernatants was assayed by an agar-diffusion method using 1.5 mg ml^−1^ l-α-phosphatidylcholine (Sigma-Aldrich S.r.l., Milano) [32]. Gels were incubated 16 h at 25°C and PC-PLC activity was quantitated by comparison of turbidity areas to those in a standard curve for pure PC-PLC (Sigma-Aldrich S.r.l.).

### Molecular typing

Randomly amplified polymorphic DNA (RAPD) fingerprinting of bacterial genomes was performed with the primers RPO2 (5′-GCGATCCCCA-3′), M13 (5′-GAGGGTGGCGGCTCT-3′), and Pro-Up (5′-GCTGCTGGCGGTGG-3′) [8], HLWL85 (5'-ACAACTGCTC-3') [34], OPE02 (5’-GGTGCGGGAA-3’), OPE03 (5’-CCAGATGCAC-3’), OPD02 (5’-GGACCCAACC-3’), and OPD03 (5’-GTCGCCGTCA-3’) [35]. PCR conditions were as follows: 30 cycles consisting of 94°C for 1 min, 36°C for 1 min, and 72°C for 2 min, followed by one cycle consisting of 72°C for 10 min [8]. The reproducibility of RAPD profiles was assessed in at least three separate experiments.

### Statistical analysis

All numerical data are expressed as mean plus standard deviations and Student’s *t*-test has been applied as statistical method.

## Results

### Identification of the spore formers contained in probiotic formulations

Considering the importance of compositional quality for commercial probiotic products and the difficulties in species identification within the genus *Bacillus*, we first analyzed the formulations reported in Table 1 searching for heat-resistant bacterial forms (i.e. spores). All morphologically different colonies isolated seeding the thermally-treated probiotic suspensions were subjected to biochemical and MALDI-TOF MS identification. Sequencing of the 16S rRNA gene, the reference technique to identify bacterial isolates, was also applied.

Table 2 summarizes the results obtained with all the identification procedures. On the basis of the sequencing results (Table 2), five (products 1, 3, 4, 5, 7) among the ten tested probiotic formulations resulted to contain spores of the *Bacillus* species declared on the labels. In four other formulations, spores of contaminant species in addition to the labeled ones were found. In particular, *Bacillus licheniformis* was recovered from product 2, *Bacillus badius* from 6, *Lysinibacillus fusiformis* from 9, and *Bacillus cereus* and *L*. *fusiformis* from 10. Formulation 8 was found to contain *B*. *subtilis* spores instead of *B*. *clausii* spores. The BCL card, which is produced for the automatic identification of the most significant aerobic endospore-forming species of the family *Bacillaceae*, was able to correctly identify all the *Bacillus* isolated strains contained in the probiotic formulations (Table 2). Five strains were identified with a very good probability of correct identification, seven with good probabilities, and one with an acceptable probability. On the other hand, the BCL kit misidentified *L*. *fusiformis* as *Brevibacillus choshinensis*. The Bruker Biotyper expresses the identification of an organism as a score based on pattern matching and considers that a score ≥ 2.00 should be obtained for correct identification at the species level. As shown in Table 2, the *B*. *badius*, *B*. *cereus*, and *B*. *subtilis* isolates were correctly identified with scores always higher than the manufacturer’s cut-off. As regards *B*. *clausii* isolates, three of the six probiotic strains gave a ≥ 2.00 score in one of the two replicates. Nevertheless, the other three strains were identified with scores ranging from 1.971–1.772 in concordance with the reference method. A similar performance of MALDI-TOF MS was observed for *L*. *fusiformis* (score range 2.073–1.763). *B*. *licheniformis* and *B*. *coagulans* isolates were never misidentified, but the score values were too low for identification at the species level (global score range 1.847–1.366). The four strains contained in formulation 4 were isolated on selective plates and subjected to MALDI-TOF MS analysis. All strains were identified as *B*. *clausii* with following scores: NR 2.121/2.011; OC 1.910/1.661; SIN 1.702/1.702; T 1.797/1.693.

**Table 2:** Identification of the spore-forming bacteria contained in each probiotic formulation.

Formulation	Labeled organisms	N. of identified species	Biochemical identification BCL(^a^)	MALDI-TOF MS identification(^b^)	16S rDNA sequencing
1	*Bacillus clausii*	1	*B*. *clausii* (94%)	*B*. *clausii* (1.953/1.934)	*B*. *clausii*
2	*Bacillus coagulans* and other bacteria^c^	2	*B*. *coagulans* (92%) *B*. *licheniformis* (90%)	*B*. *coagulans* (1.431/1.366) *B*. *licheniformis* (1.847/1.732)	*B*. *coagulans* *B*. *licheniformis*
3	*Bacillus clausii*	1	*B*. *clausii* (89%)	*B*. *clausii* (1.841/1.772)	*B*. *clausii*
4	*Bacillus clausii*	1	*B*. *clausii* (95%)	*B*. *clausii* (2.121/1.661)	*B*. *clausii*
5	*Bacillus clausii*	1	*B*. *clausii* (93%)	*B*. *clausii* (2.120/1.913)	*B*. *clausii*
6	*Bacillus coagulans* and other bacteria^d^	2	*B*. *coagulans* (92%) *B*. *badius* (90%)	*B*. *coagulans* (1.635/1.505) *B*. *badius* (2.226/2.095)	*B*. *coagulans* *B*. *badius*
7	*Bacillus coagulans*	1	*B*. *coagulans* (94%)	*B*. *coagulans* (1.745/1.529)	*B*. *coagulans*
8	*Bacillus clausii*	1	*B*. *subtilis* (90%)	*B*. *subtilis* (2.216/2.184)	*B*. *subtilis*
9	*Bacillus clausii*	2	*B*. *clausii* (88%) *Brevibacillus choshinensis* (96%)	*B*. *clausii* (2.140/1.868) *Lysinibacillus fusiformis* (1.970/1.867)	*B*. *clausii* *L*. *fusiformis*
10	*Bacillus clausii*	3	*B*. *clausii* (93%) *B*. *cereus* (89%) *B*. *choshinensis* (96%)	*B*. *clausii* (1.971/1.815) *B*. *cereus* (2.207/2.196) *L*. *fusiformis* (2.073/1.763)	*B*. *clausii* *B*. *cereus* *L*. *fusiformis*

Labeled organisms and identification by biochemical tests, MALDI-TOF MS, and 16S rDNA sequencing of each bacterial isolate contained in the analyzed formulations.

^a^ Probability of correct identification.

^b^ Identification scores of the two replicates.

^c^ *Lactobacillus rhamnosus* and *Lactobacillus helveticus*.

^d^ *Lactobacillus acidophilus* and *Bifidobacterium animalis* ssp. *lactis*.

### Enumeration of the organisms contained in probiotic formulations

Table 3 reports the labeled number of bacteria belonging to the *Bacillus* genus, the total counts (total CFU), and the counts of spores (CFU from spores only) obtained for a unit dose of each product after plate counting on TSH medium. Total CFU were concordant with the labeled number of cells for products 1 and 4. Formulations 2, 3, 5, 6, and 7 produced a lower CFU number per unit dose than that declared by the manufacturers. Total CFU originating from products 8, 9, and 10 were 1–3 log higher than those labeled.

**Table 3:** Enumeration of the spore formers contained in a unit dose of each probiotic formulation.

**Formulation**	**Unit dose**	**Labeled *Bacillus* no.**	**Total CFU**	**CFU from spores only**
1	1 vial	2 × 10^9^	1.4 ± 1.1 × 10^9^	5.9 ± 5.0 × 10^9^
2	1 bottle	4.55 × 10^9^	5.6 ± 0.5 × 10^4^	5.5 ± 1.5 × 10^4^
3	1vial	4 × 10^9^	2.8 ± 2.2 × 10^7^	1.1 ± 0.5 × 10^7^
4	1 vial	2 × 10^9^	1.2 ± 0.9 × 10^9^	1.7 ± 0.7 × 10^9^
5	1 vial	2 × 10^9^	3.7 ± 2.3 × 10^6^	3.4 ± 2.1 × 10^6^
6	1 capsule	5 × 10^9^	4.8 ± 3.2 × 10^4^	2.0 ± 1.7 × 10^5^
7	1 bottle	3 × 10^9^	1.5 ± 1.5 × 10^8^	1.1 ± 5.8 × 10^7^
8	1 bottle	1 × 10^10^	4.3 ± 2.9 × 10^11^	1.6 ± 1.4 × 10^12^
9	1 bottle	1 × 10^10^	7.1 ± 1.5 × 10^12^	9.0 ± 0.5 × 10^12^
10	1 bottle	5 × 10^9^	4.2 ± 3.1 × 10^12^	1.9 ± 1.1 × 10^12^
		**Identified species**		
2	1 bottle	*B*. *coagulans* *B*. *licheniformis*	1.2 ± 0.8 × 10^4^ 6.0 ± 3.2 × 10^3^	4.3 ± 0.8 × 10^4^ 8.5 ± 1.5 × 10^3^
6	1 capsule	*B*. *coagulans* *B*. *badius*	1.5 ± 1.3 × 10^4^ 3.5 ± 1.5 × 10^3^	2.0 ± 1.7 × 10^5^ 9.0 ± 3.7 × 10^2^
9	1 bottle	*B*. *clausii* *L*. *fusiformis*	6.4 ± 0.9 × 10^12^ 7.0 ± 0.7 × 10^11^	6.5 ± 0.4 × 10^12^ 2.5 ± 0.2 × 10^12^
10	1 bottle	*B*. *clausii* *B*. *cereus* *L*. *fusiformis*	1.8 ± 1.1 × 10^12^ 1.0 ± 0.6 × 10^10^ 1.3 ± 0.3 × 10^11^	3.4 ± 1.6 × 10^12^ 2.5 ± 0.5 × 10^10^ 7.0 ± 1.0 × 10^11^

Enumeration of the spore formers on TSH medium. For products containing more than one isolate, separate counts are reported.

The contaminant microorganisms present in products 2, 6, 9, and 10 were recovered at high levels, comparable to the labeled *Bacillus* species (Table 3, lower part). Interestingly, a Gram-negative organism was also isolated on TSH plates (1.2 ± 0.3 × 10^11^ total CFU/unit dose) from product 8. The strain was identified as *Acinetobacter baumannii* by MALDI-TOF MS (scores 2.474/2.302).

Since *B*. *cereus* is well known as food pathogen, able to cause diarrheal and emetic syndromes, and exhibits virulence in a strain specific manner, the potential pathogenicity of the *B*. *cereus* strain isolated from formulation 10 was evaluated. S1 Fig shows the amplification profile of *B*. *cereus* virulence genes from this isolate (S lanes). The *B*. *cereus* ATCC 14579 reference strain was used as positive control for PCR amplification (C+ lanes). The *sph*, *entT*, *entS*, *plcA*, and the three *nhe* genes were found present in the isolated strain. Moreover, by an agar diffusion assay, we demonstrated that the *B*. *cereus* isolate secretes 0.04 Uml^-1^ of PC-PLC, an amount comparable to a low PC-PLC producer [32]. All together, these data indicate that the *B*. *cereus* strain isolated from product 10 is potentially toxic for humans.

### Molecular typing of *B*. *clausii* and *B*. *coagulans* isolates

With the aim of evaluating whether the different probiotic formulations contained an identical *B*. *clausii* or *B*. *coagulans* strain, molecular typing of the isolates of these species was performed by RAPD-PCR. This kind of whole genome fingerprinting has successfully been used to differentiate strains belonging to the genus *Bacillus* [8, 35, 36].

**Fig 1:**
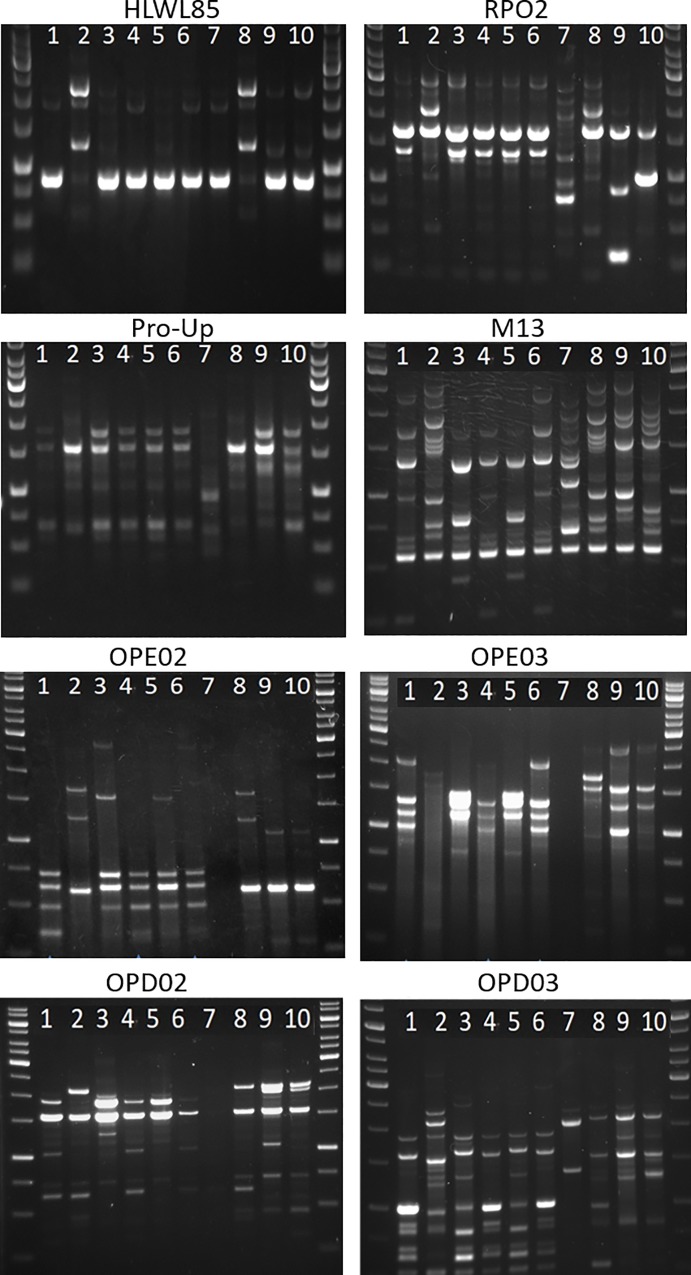
RAPD-PCR amplification obtained with different primers from *B*. *clausii* strains. Used primer: HLWL85, RPO2, Pro-Up, M13, OPE02, OPE03, OPD02, OPD03. 1: strain OC from formulation 4; 2: strain from formulation 10; 3: strain from formulation 9; 4: strain from formulation 5; 5: strain from formulation 3; 6: strain from formulation 1; 7: ATCC 10317; 8: DSM 8716; 9: ATCC 21536; 10: ATCC 21537.

**Fig 2:**
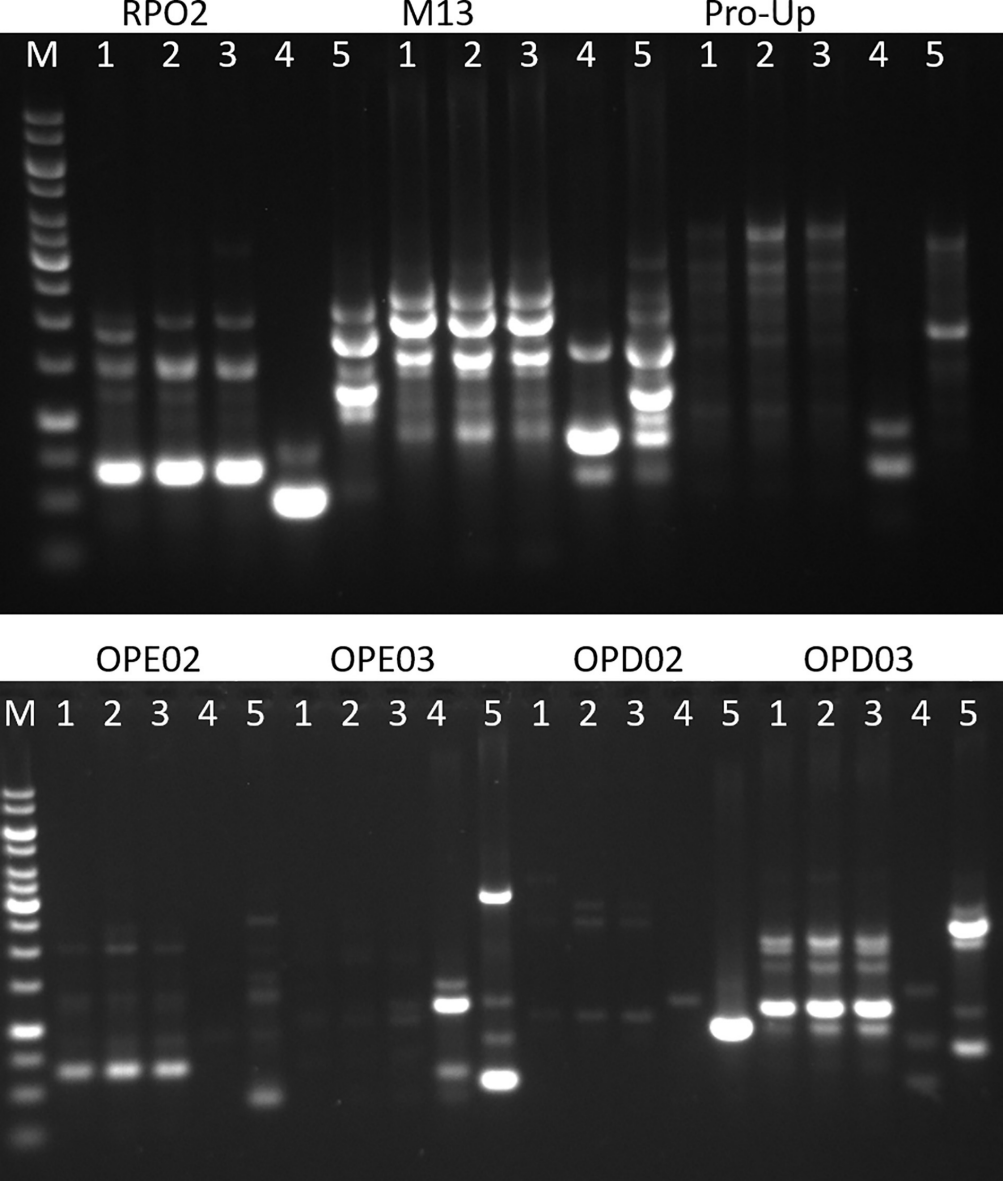
RAPD-PCR amplification obtained with different primers from *B*. *coagulans* strains. Used primer: RPO2, M13, Pro-Up, OPE02, OPE03, OPD02, OPD03. 1: strain from formulation 2; 2: strain from formulation 6; 3: strain from formulation 7; 4: FLtas1; 5: FP22.

In this study, different primers were used to amplify the genomic DNA of the *B*. *clausii* (Fig 1) and *B*. *coagulans* isolates (Fig 2). Obtained profiles were compared with those produced from *B*. *clausii* (Fig 1) and *B*. *coagulans* collection strains (Fig 2). Since previous studies demonstrated the identical profiles obtained by RAPD-PCR amplification of the four *B*. *clausii* strains [8], herein we used strain OC as representative of this probiotic formulation.

Global analysis of the generated amplification patterns revealed genomic similarities between i) the strain contained in formulation 10 and *B*. *clausii* DSM 8716; ii) the strains contained in the formulations 1, 4, and at less extent 5; and iii) the strains contained in the formulations 9 and 3. As regards *B*. *coagulans* strains, the three probiotic isolates showed similar amplification profiles. Nevertheless, the strain isolated from formulation 2 produced characteristic bands when the primers RPO2 and OPD02 were used, thus indicating genomic divergences from the other strains.

## Discussion

Quality control of probiotic products is the focus of numerous organizations worldwide, with the European Society for Pediatric Gastroenterology Hepatology and Nutrition recently highlighting the importance of a more stringent control of commercialized probiotic products [37]. As established in the Food and Agriculture Organization (FAO) and the World Health Organization (WHO) guidelines, for being used as probiotic it is essential that the organism is considered as GRAS (generally recognized as safe) [38]. The European Food Safety Authority (EFSA) has developed a list of safe biological agents defined as QPS (qualified presumption of safety) for pre-market safety assessment. In 2018, the Italian Ministry of Health published its guidelines on probiotics in which the assessment of the taxonomic position is considered a crucial point to guarantee safety of the used microorganism [39].

The probiotic market is continuously expanding and new products are constantly developed. Formulations are mainly constituted by lactobacilli, bifidobacteria, spore formers, or yeast. In the global nutraceutical and pharmaceutical market, the spore-containing probiotics based on *Bacillus* spp. are making a major contribution [6] and, in some countries, have a long tradition of use. The genus *Bacillus* has undergone considerable taxonomic changes and different genetic approaches and biological assays have been developed for differentiating more than 300 species belonging to this genus [40, 41].

Optimizing the identification procedures for probiotic *Bacillus* strains is essential for the quality control of preparations containing bacterial spores. Three methods based on different approaches (biochemical/metabolic, proteomic, and genetic) were used in this study to identify *Bacillus* strains contained in probiotic formulations. Our overall results indicate that the biochemical BCL card test performed well in identifying all the *Bacillus* probiotic strains. Only the contaminants isolated from the formulations 9 and 10 were identified as *B*. *coshinensis* instead of *L*. *fusiformis*. The results obtained with MALDI-TOF MS were always concordant with 16S rDNA sequencing. Nevertheless, the obtained scores were sometimes lower than the values suggested by the manufacturer’s guidelines for correct species identification, especially when dealing with *B*. *coagulans* strains [16]. However, different replicates always produced correct identification, thus suggesting that even low score values could be considered as accurate for the *Bacillus* species most commonly present in probiotic formulations.

As regards the quality of the analyzed formulations, all the labeled *Bacillus* species were recovered, with the only exception of product 8 that includes *B*. *subtilis* instead of *B*. *clausii*. However, contaminant microorganisms were frequently observed. Formulations 2, 6, 9, and 8 contained one contaminant organism and even two additional species were found in product 10. These results raise some concern about quality control in the procedures during preparation of these formulations.

It appears of relevance the finding that most of the isolated contaminants may behave as human pathogens. *B*. *licheniformis* is increasingly recognized as cause of serious diseases such as bacteremia, peritonitis, food poisoning and eye infections mainly in immunocompromised patients [42–45]. *L*. *fusiformis*, typically isolated from different environments, has occasionally been reported as opportunistic pathogen [46]. *A*. *baumannii* is an opportunistic nosocomial pathogen responsible for a vast array of infections with high mortality rate and one of the six most important multidrug-resistant microorganisms in hospitals worldwide. Notably, gut colonization with *A*. *baumannii* has been demonstrated to frequently precede bacteremia in critically ill patients [47]. *B*. *cereus* is well known to cause foodborne intoxications as well as local and systemic infections in humans [42, 48]. The pathogenic potential of this bacterium is related to the secretion of several virulence proteins such as hemolysins, phospholipases, trimeric toxins (HBL and NHE), and CytK [48–50]. The finding that the *B*. *cereus* strain contained in formulation 10 has the potential to produce many virulence factors highlights that the presence of this food pathogen in a probiotic formulation is far for being of negligible importance.

The number of viable cells contained into a probiotic formulation is one of the qualifications that the FAO and the WHO document have recommended [38]. The Italian Ministry of Health guidelines indicate that the minimal amount of a probiotic to be active is 1 × 10^9^ CFU per day [39] since lower numbers of microorganisms could preclude an effective health benefit. In our study, the number of viable cells of the species declared to be contained in the analyzed formulations most often do not comply with the label and the Italian guideline requirements.

The confirmed identity of the microorganism, not only at the species level, but also at the strain level is a prerequisite to ensure that a commercial product will deliver the claimed beneficial health effect. Molecular typing of the most common *Bacillus* species contained in the tested probiotic Italian products (*B*. *clausii* and *B*. *coagulans*) indicates the presence of different strains. Therefore, the demonstrated efficacy for one product cannot be automatically translated to another product containing the same *Bacillus* species.

In conclusion, only two of the ten analyzed formulations (1 and 4) qualitatively and quantitatively respect what is on the label, thus suggesting that probiotic products should have a more stringent quality control process that ensures contents match what is on the label. By the way, only these two formulations are registered as medicinal products and therefore subjected to more rigorous quality controls. As regards formulations containing *Bacillus* spores, quality controls require trained personnel able to morphologically recognize different bacterial colonies and modern technologies for the identification of these bacteria.

## Supporting information

S1 Fig Amplification profiles of toxin-encoding genes from *B*. *cereus*. S lanes: *B*. *cereus* strain isolated from formulation 10. C+ lanes: *B*. *cereus* ATCC 14579 reference strain.
(TIF)
