# Supplementary figures and images for: Identification of Bacillus species: Implication on the quality of probiotic formulations
Source: PLoS One. 2019 May 20;14(5):e0217021. doi: 10.1371/journal.pone.0217021 (PMC6527297; doi:10.1371/journal.pone.0217021)

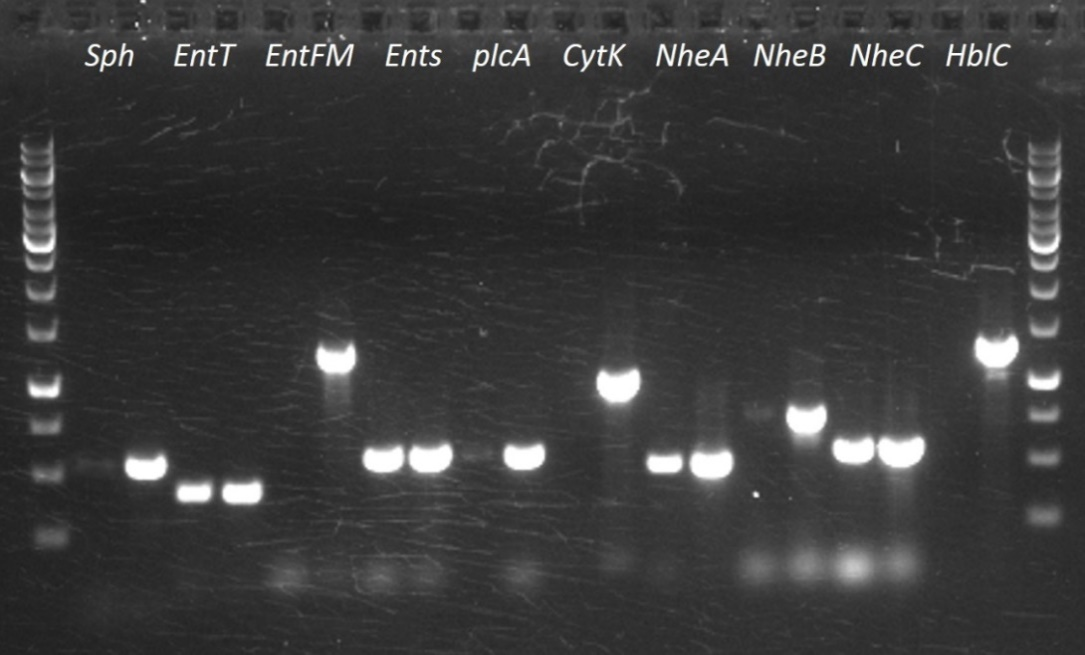

Supplement: S1 Fig — S lanes: B. cereus strain isolated from formulation 10. C+ lanes: B. cereus ATCC 14579 reference strain. (TIF) [file pone.0217021.s001.tif]
